# Atypical Clinical Presentation of Xanthogranulomatous Pyelonephritis in a Female Child: A Case Report

**DOI:** 10.7759/cureus.53666

**Published:** 2024-02-05

**Authors:** Kamel A Alenazi

**Affiliations:** 1 Department of Pediatrics, Imam Mohammad Ibn Saud Islamic University, Riyadh, SAU

**Keywords:** children, flank pain, proteus mirabilis, chronic obstructive renal suppuration, xanthogranulomatous pyelonephritis

## Abstract

Xanthogranulomatous pyelonephritis (XGPN) is an uncommon chronic obstructive renal suppuration disease. Histopathologically, XGPN manifests as lipid-laden macrophage infiltration in renal microstructure and inflammation of an engorged non-functional kidney. Nephrectomy is the standard therapeutic treatment, and the overall prognosis is good. Here, we report a case of XGPN presented as flank pain in an otherwise healthy child.

## Introduction

Xanthogranulomatous pyelonephritis (XGPN) is an extremely rare chronic obstructive, typically unilateral, renal suppurative granulomatous infection, which injures glomerular and periglomerular microstructure along with renal parenchyma [1]. XGPN is a disease that classically affects the female gender of the middle-aged group [1]. Seldom diagnosed in the pediatric cohort, children account for 16% of the nephrectomy specimens [2], and its etiology is attributed to urinary tract obstruction typically from infections and inborn urological deformities, such as frequent urinary tract infections (UTIs), nephrolithiasis, and posterior urethral valve [3]. Non-specific symptoms and radiological resemblance of renal mass impinging nearby structures, like that of renal cancer, often result in misdiagnosis or delay in appropriate therapeutic management [4]. Antibiotic treatment and radical nephrectomy make the standard therapeutic regime for this occasional urological condition [3]. Here, we report a case of an 11-year-old girl who was admitted to King Fahad Medical City, Riyadh, Saudi Arabia, with suspicion of UTI. During her workup and management, she underwent initial percutaneous drainage for the right perinephric collection and finally a successful right radical nephrectomy after re-accumulation of the right psoas abscess, which is a very uncommon and atypical presentation. The histopathological examination of the resected kidney tissue revealed XGPN.

## Case presentation

An 11-year-old girl presented to the emergency department with a history of fever for six weeks, ranging from 38°C to 39°C, measured axillary and responding well to antipyretic. Her parents documented one to two fever spikes per day accompanied by on/off mild right flank pain; however, there was no history of chills, loss of appetite, or weight loss. Despite fever, the patient was very active. Her medical and family history was insignificant except for her father's history of resolved tuberculosis two years back. Physical examination was also unremarkable. According to the parents, they sought medical advice in different clinics and received multiple courses of antibiotics, including amoxicillin/clavulanic acid, cefuroxime, and trimethoprim/sulfamethoxazole. However, she did not complete the entire course of antibiotics most of the time and showed no signs of improvement. Table 1 presents the patient's laboratory investigations.

**Table 1:** Laboratory investigations of the patient

Laboratory parameters	Findings	Reference range
Hemoglobin	9.6 g/dL	11–15 g/dL
Platelets	569 × 10^9^/L	150–450 × 10^9^/L
White blood cells	14.5 × 10^9^/L	4.3–11.3 × 10^9^/L
Neutrophils	87%	30–70%
Lymphocytes	14.2%	25–75%
Erythrocyte sedimentation rate	120 mm/hr	0–20 mm/hr
Brucella	Negative	Negative
Cytomegalovirus	Negative	Negative
Epstein-Barr virus	Negative	Negative

The patient was admitted, and the initial diagnosis was a UTI. An abdominal ultrasound was then requested, on which the right ureteric jet was not noticeable, which indicated possible obstruction of the right kidney with superadded infection. Moreover, compensatory enlargement of the otherwise normal left kidney was observed. The patient was immediately started on intravenous (IV) ceftriaxone. The teams of Urology (for surgical intervention, such as nephrectomy, and assessment of anatomical factors contributing to recurrent infections), Nephrology (for renal function, complication management, and postoperative care), and Infectious Diseases (for identifying causative bacteria, guiding antibiotic therapy, and preventing infection recurrence) were consulted. This collaborative approach ensures a comprehensive strategy for diagnosis, treatment, and long-term management, enhancing overall patient outcomes and minimizing complications associated with this complex renal condition. The impressions included a partially treated UTI with pyelonephritis (Nephrology and Infectious Diseases) and infected cyst versus pyelonephritis secondary to vesicoureteral reflux/ureterovesical junction obstruction (Urology). The teams of Urology, Nephrology, and Infectious Diseases advised to perform abdominal contrast-enhanced computed tomography (CT) scan to look for any deep abscess that might need urgent percutaneous drainage by interventional radiology.

**Figure 1:**
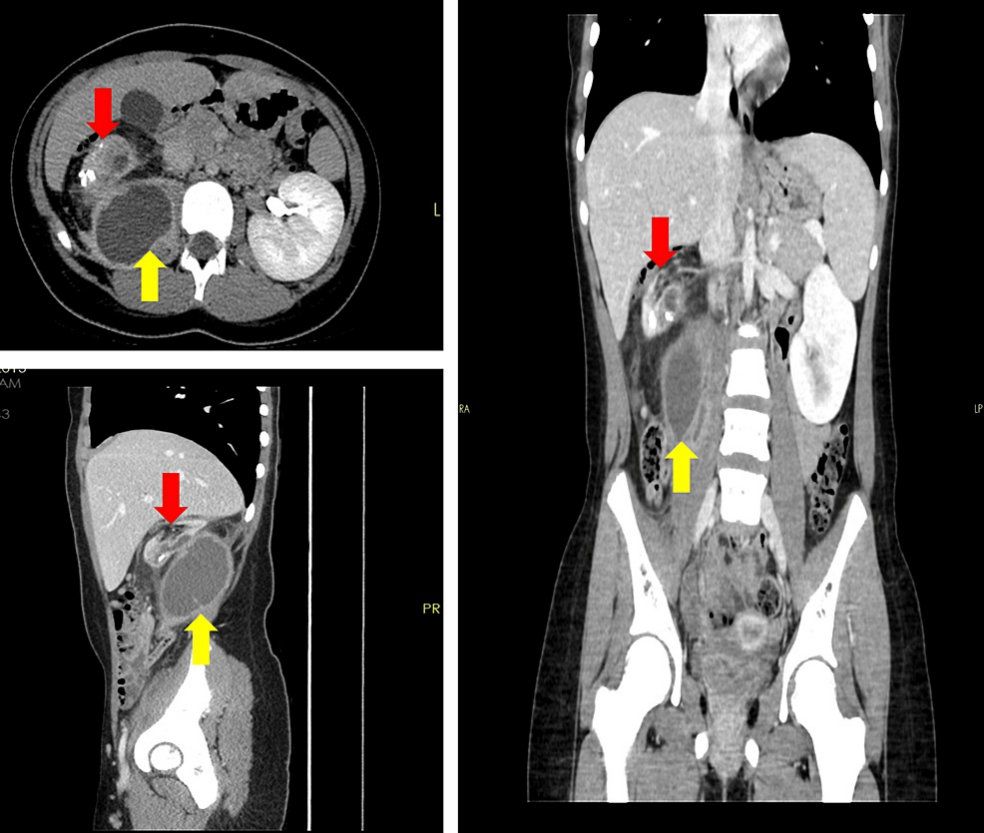
Contrast-enhanced abdominal computed tomography (CT) scan showing global destruction of the right kidney with extensive stones, perinephric/paranephric diffuse inflammatory changes, multiple spots of calcifications, and a large well-defined right psoas abscess. The red arrow represents a destroyed kidney with stones, and the yellow arrow shows pus collection.

Abdominal contrast CT findings are depicted in Figure 1. The CT scan was conclusive of a globally destroyed right kidney with replacement of its parenchyma with multiple low attenuation non-enhancing tissue. There are numerous large stones with several spots of calcifications and obvious perinephric and paranephric fat stranding. Furthermore, a large, well-defined right psoas fluid collection with wall enhancement represents an abscess. Based on the CT findings, the differential diagnosis included tuberculous pyelonephritis or XGPN.

A treatment plan of percutaneous drainage (to successfully resolve the perinephric pus collection), IV ceftriaxone administration in conjunction with drainage (to resolve right psoas abscess), abscess culture (to rule out specific infections), IV metronidazole and continued IV ceftriaxone (to contribute to symptom improvement), cardiology consultation (to rule out cardiac vegetation via echocardiography), and a structured antibiotic regimen of a two-week course of IV ceftriaxone followed by oral metronidazole and cefuroxime (to ensure sustained recovery) was devised.

The patient underwent interventional radiology for percutaneous drainage of the right perinephric pus collection under the standard sterile procedure. An 8-French multipurpose drainage catheter was carefully inserted into the abscess cavity using a coaxial system, and 150 ml of clear pus was aspirated immediately and sent for further analysis. Post-procedural scan demonstrated no signs of active bleeding or significant hematoma. She was kept on IV ceftriaxone, and an ultrasound was advised two days later, which showed a resolution of the right psoas abscess. The abscess culture report was negative for *Mycobacterium tuberculosis* anaerobes and fungal infection.

Infectious Diseases reviewed the patient's status post-drainage, added IV metronidazole and continued IV ceftriaxone, and advised consulting the cardiology to rule out any vegetation. Echocardiography showed a normal cardiac structure and function with no evidence of vegetation. The patient completed a two-week course of IV ceftriaxone, and her fever and flank pain improved during her hospital stay. She was discharged home on oral metronidazole for three more weeks, followed by two weeks of oral cefuroxime, and counseled to follow up as an outpatient.

On follow-up with Infectious Diseases, the patient completed a five-week antibiotic course, was afebrile, and was doing fine. Her complete blood count improved, and her urine culture was also negative. Upon follow-up with Urology, an ultrasound of the abdomen and dimercaptosuccinic acid (DMSA) were done to check for any recollection of the abscess. Abdominal ultrasound findings were compared with previous ultrasound scans. Mild interval improvement of the previously seen collection/inflammatory lesion in the right renal fossa/psoas muscle was seen with compensatory enlargement of the otherwise normal left kidney. The DMSA scan also revealed compensatory hypertrophy of the left kidney with a non-visualized and non-functioning right kidney.

After six months of drainage, the patient presented to the general Pediatrics and Urology clinic with subjective fever and right flank pain radiating to the groin, increased when lying down and relieved by analgesics with no urinary symptoms. Repeated laboratory investigation demonstrated raised erythrocyte sedimentation rate (ESR). The findings were compared with earlier CT examinations and revealed an interval increase in the size of the right psoas abscess by more than 50%. The patient was once again admitted under urology care as a case of re-accumulation of the right psoas abscess for drainage and nephrectomy.

The patient underwent interventional radiology for drainage of the right perinephric abscess under anesthesia with ultrasound guidance. An 8-French multipurpose drainage catheter was inserted into the right nephric collection, and 200 ml of pus was aspirated. The abscess was sent for culture, which showed *Proteus mirabilis* (gram-negative, rod-shaped, and anaerobic bacteria). An open laparotomy was performed, her right kidney was resected, and the patient was kept on IV cefuroxime for one week. Renal tissue was then sent for histopathology analysis, which confirmed the XGPN diagnosis.

The patient made an uneventful recovery and was discharged. On discharge, the parents were informed regarding the solitary kidney and stringently instructed to avoid nephrotoxic medications. The patient improved remarkably post-nephrectomy. However, two weeks later, she developed postoperative wound infection from extended-spectrum beta-lactamase (ESBL) producing *Escherichia coli*. She received ciprofloxacin for three weeks and continued to follow up with Infectious Diseases as an outpatient. At the two-week follow-up, the patient was asymptomatic and afebrile with a clean surgical site. There was no fluid discharge, and no tenderness was witnessed on examination. Abdominal ultrasonography showed no evidence of fluid or pus collection in the right renal area, right lumbar region, and right iliac fossa. The parents were encouraged to continue follow-up every six months and are thus far compliant. The patient is doing fine with no complaints of symptoms remission.

## Discussion

XGPN is a very uncommon chronic obstructive renal suppuration disease that histopathologically manifests as lipid-laden macrophage infiltration in the renal microstructure and an inflamed and engorged non-functional kidney [5]. The exact etiological risk factor remains obscured. It is frequently linked with bacterial superinfections like *Escherichia coli*, *Proteus mirabilis*, and, every now and then, *Pseudomonas* species [6]. Reportedly, diabetes mellitus, renal calculi, and immunocompromised health state could be predisposing factors to this rare condition [7]. Children with congenital malformations in the urinary tract are more likely to develop XGPN, as suggested in the literature [8]. XGPN is often mistaken as a renal neoplasm owing to its local invasion of nearby structures like the pancreas, duodenum, spleen, and the psoas muscle.

XGPN frequently involves unilateral kidneys, as was the case for our patient. However, reports of bilateral kidney involvement have also been communicated [9,10]. It is common among middle-aged women [11]; however, it is rarely documented in children and of the female gender [4]. In fact, the left kidney is more prone to get affected in children, and we witnessed the opposite case (right kidney involvement) in our female patient [4]. The precise pathological phenomenon is still uncertain, but a general consensus is that long-standing kidney obstruction with superimposed infection by *Escherichia coli*, *Proteus mirabilis*, and *Pseudomonas* species could ensue the XGPN disease process [4]. Our patient had a superimposed infection with *Proteus mirabilis*.

While imaging modalities such as ultrasound can spot XGPN, contrast-enhanced CT and magnetic resonance imaging (MRI) offer high sensitivity and detect swollen non-functional kidney +/- invasion to nearby tissues [12,13]. In our patient, abdominal contrast-enhanced CT findings revealed a globally destroyed right kidney with numerous large renal stones, a classic characteristic that is documented in approximately 80% of the XGPN cases [4]. Moreover, multiple spots of calcifications with perinephric and perinephric fat stranding and a large well-defined right psoas abscess collection were also found. XGPN is often regarded as the pseudotumor due to its invasive propensity toward neighboring structures [14]. This was the case in our patient, as it was evidenced through contrast CT by the involvement of the psoas muscle and, subsequently, psoas abscess fluid collection.

Our patient manifested classical clinical UTI signs and symptoms of long-standing fever and flank pain [15] with a history of multiple incomplete courses of antibiotics. Our patient did not have any congenital urinary tract malformation that one would have expected in this case scenario. Histopathologically, XGPN appears as lipid-laden macrophage infiltration with a blend of immune-inflammatory cells [5], which, as expected, was found to be characteristic of XGPN in our patient. Anemia with elevated erythrocyte sedimentation rate and leukocytosis are common non-specific laboratory findings. In unilateral XGPN, serum creatinine and urea are often within normal range, and they get deranged only in bilateral renal involvement cases due to the destruction of both kidney tissues. Urinalysis, including culture, shows proteinuria, leukocytosis, and bacterial growth, which assists in determining antibiotic sensitivity [16].

Nephrectomy of the non-functional kidney remains the mainstay for absolute diagnosis and cure of XGPN. Nephron-sparing surgery could also be an option, but only in circumstances of bilateral kidney involvement demonstrating substantial residual function in kidney tissues [17]. Our patient also underwent open, laparoscopic right kidney nephrectomy, and the histopathological and culture examination of the resected kidney tissue revealed XGPN and *Proteus mirabilis* organisms, respectively.

It is suggested that antibiotic therapy has only been impactful for XGPN in a few cases, but a temporary course could be administered pre- and post-nephrectomy as prophylaxis. The selection of antibiotics can be personalized based on culture and sensitivity results. However, empiric antibiotic therapy can be started given the sensitivity of species to specific antibiotics like *Proteus* species and *Escherichia coli* to first-generation cephalosporins and *Pseudomonas* species to third-generation cephalosporins, aminoglycosides, or fluoroquinolones [18]. While there are no specific guidelines for the duration of the antibiotic course, case reports and case series data suggest a one-week course of antibiotics post-nephrectomy [4,19]. Post-nephrectomy, IV cefuroxime was continued for one week in our patient. However, two weeks later, she developed an ESBL-producing *Escherichia coli* post-operative wound infection. She was then given oral ciprofloxacin for three weeks. Upon follow-up and during the submission of this manuscript, the patient remained asymptomatic and afebrile with no fluid discharge and tenderness and is doing absolutely fine.

The clinical management of XGPN presents challenges due to its diagnostic complexity, multidisciplinary coordination, and potential complications from surgical interventions. To address these, clinical practice should prioritize enhanced awareness through continuous education, establish standardized protocols for a multidisciplinary approach, and develop comprehensive postoperative care plans. Patient education on follow-up importance and recognizing early complications will empower individuals in their healthcare journey. Implementing these strategies will contribute to improved outcomes and a more streamlined approach to dealing with the complexities of XGPN in clinical practice.

## Conclusions

XGPN signifies a rare and complex clinical entity described by its inflammatory infiltration, mimicking neoplastic processes, and often causing substantial destruction of renal tissue. This case stresses the diagnostic intricacy surrounding XGPN, its diverse clinical presentation across age brackets and genders, and the requirement for a multidisciplinary approach for timely diagnosis and management. The clinical course of our case highlights the value of timely nephrectomy in unilateral cases and the cautious use of well-aimed antibiotic therapy, contemplating the sensitivity patterns of identified bacterial species. Notwithstanding the postoperative complication, effective management led to the patient's uneventful and complete recovery, accentuating the significance of attentive follow-up in warranting favorable outcomes for individuals affected by this uncommon renal condition. Recommendations for future cases involve enhancing awareness among healthcare professionals about the diverse presentations of XGPN and promoting collaboration among specialists for a comprehensive diagnostic and therapeutic strategy. In addition, further research into the optimal management approaches, including antibiotic selection and postoperative care, would contribute to refining the treatment of this challenging clinical entity.
